# Tomographic analysis of midpalatal suture prior to rapid maxillary expansion

**DOI:** 10.1590/2177-6709.26.3.e2119300.oar

**Published:** 2021-06-30

**Authors:** Ilana Oliveira CHRISTOVAM, Cinthia de Oliveira LISBOA, Giselle Naback Lemes VILANI, Roberto Carlos Bodart BRANDÃO, Maria Augusta Portella Guedes VISCONTI, Claudia Trindade MATTOS, Antônio Carlos de Oliveira RUELLAS

**Affiliations:** 1 Universidade Federal do Rio de Janeiro, Faculdade de Odontologia, Departamento de Ortodontia (Rio de Janeiro/RJ, Brazil).; 2 Universidade Federal Fluminense, Departamento de Clínicas Odontológicas (Niterói/RJ,Brazil).; 3 Private practice (Belo Horizonte/MG, Brazil).; 4 Universidade Federal do Espírito Santo, Faculdade de Odontologia, Departamento de Ortodontia (Vitória/ES, Brazil).; 5 Universidade Federal do Rio de Janeiro, Departamento de Patologia e Diagnóstico Oral (Rio de Janeiro/RJ, Brazil).; 6 Universidade Federal Fluminense, Faculdade de Odontologia, Departamento de Ortodontia (Niterói/RJ,Brazil).

**Keywords:** Palatal expansion technique, Imaging, three-dimensional, Cone-beam computed tomography

## Abstract

**Introduction:**

In Orthodontics and Facial Orthopedics, the timing of treatment onset may be critical and individual analysis should be applied to promote a favorable treatment planning. In this study, individual analysis of midpalatal suture (MS) and palatal measurements were performed in teenagers and young adult patients treated with rapid maxillary expansion (RME).

**Description:**

Twenty-six patients submitted to RME with a tooth-supported appliance (Hyrax) were evaluated. The inclusion criteria were: minimum age of 14 years, presenting all posterior teeth, diagnosed with transverse maxillary discrepancy, and with a clinical indication for maxillary expansion. The pretreatment CBCT scans of these patients were assessed to obtain the stages of MS maturation (MSM); density ratio (MSD); and palatal length, thickness (anterior, intermediate and posterior) and sagittal area.

**Results:**

The maturation stages present were C, D or E; the density ranged from 0.6 to 1, and lower density (MSD < 0.75) and higher density (MSD ≥ 0.75) groups were determined. Individuals with higher MSD presented smaller sagittal area, compared to the lower density group. Individuals in D and E MSM stages presented smaller sagittal area and intermediate thickness, compared to stage C.

**Conclusions:**

Smaller palatal sagittal area was observed in the high MSD groups and in the stages D and E of MSM.

## RESUMO

**Introdução:**

Em Ortodontia e Ortopedia Facial, o momento de início do tratamento pode ser crítico, e uma análise individual deve ser aplicada para promover um planejamento de tratamento favorável. No presente estudo, foram realizadas a avaliação individualizada da sutura palatina mediana (SPM) e medições no palato de adolescentes e adultos jovens tratados com expansão rápida da maxila (ERM).

**Descrição:**

Foram avaliados vinte e seis pacientes submetidos à ERM com aparelho dentossuportado (Hyrax). Os critérios de inclusão foram: idade mínima de 14 anos, apresentando todos os dentes posteriores, diagnosticado com discrepância transversa da maxila e com uma indicação clínica para expansão maxilar. A tomografia computadorizada de feixe cônico (TCFC) pré-tratamento desses pacientes foi avaliada para obter os estágios de maturação da SPM (MSPM), densidade da SPM (DSPM), comprimento do palato, espessura (anterior, intermediária e posterior) e área sagital.

**Resultados:**

Os estágios de maturação presentes foram C, D ou E; a densidade variou de 0,6 a 1, e foram determinados grupos de baixa (DSPM < 0,75) e alta densidade (DSPM ≥ 0,75). Indivíduos com maior DSPM apresentaram menor área sagital, em comparação com o grupo de densidade mais baixa. Indivíduos nos estágios D e E de MSPM apresentaram menor área sagital e espessura intermediária, comparados aos indivíduos no estágio C.

**Conclusão:**

Uma menor área sagital palatina foi observada nos grupos de alta DSPM e nos estágios D e E de MSPM.

## INTRODUCTION

The ideal moment for orthodontic treatment varies according to each patient’s malocclusion. Transverse discrepancies should be treated as soon as possible,^11^ since the timing of treatment onset may be critical when treatment is implemented too late.^2^

In rapid maxillary expansion (RME), the skeletal effect is expected to be greater than the dental one; therefore, the maxillary arch width increase must be achieved by opening the midpalatal suture (MS), and not by the inclination of posterior teeth.^3,4^ However, the resistance of the suture to opening increases as suture fusion advances, which makes the RME controversial in young adults.^4^ ^,^ ^5^

The ossification of the MS occurs from the posterior to the anterior region;^6^ and is not directly related to chronological age.^7,8^ There is a consensus that RME in patients up to 14 years of age is predictable, but individual variations in MS fusion process must be analyzed based on the definition of its maturation stage (MSM)^7,9^ and density (MSD).^10^ Suture images can be obtained from cone beam computed tomography (CBCT), and that approach has been increasingly used in orthodontics.^11^

A recent study^12^ suggested that patients older than 15 years of age have a positive prognosis for RME when the MS is at an intermediate stage of maturation, although the efficacy of the MSM analysis is not conclusive to predict the magnitude of expected changes.^13^

Therefore, the purpose of the present study was to assess whether palatal baseline measurements differ in teenagers and young adult patients submitted to RME, according to their MS density ratio and maturation stage. The null hypothesis was that there is no difference.

## MATERIAL AND METHODS

The protocol of this research was approved by the Research Ethics Committee of the Federal University of Rio de Janeiro (UFRJ, protocol #68388017.5.0000.5257). 

In a previous pilot study, the area of the palate was evaluated in the sagittal section of the images of ten patients randomly chosen. The mean and standard deviation of the areas found were calculated. A sample size calculation was performed, considering a test power of 80%, α = 0.05 and a difference to be detected of 45 mm^2^, and a total of thirteen patients were required in each group.

**Figure 1:**
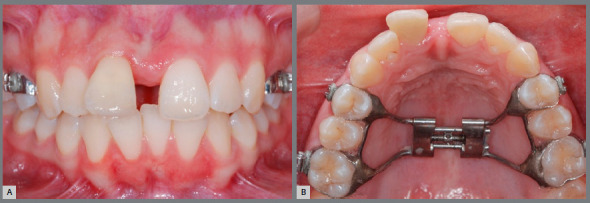
Interincisor diastema after RME with Hyrax. **A**) Frontal view; **B**) Occlusal view.

Inclusion criteria were: patients with a minimum age of 14 years, presenting all posterior teeth, diagnosed with transverse maxillary discrepancy, and who had a clinical indication for maxillary expansion. Patients were recruited for RME with a tooth-supported appliance (Hyrax), and obtained MS opening after the active phase. Two orthodontists treated the study patients in a private clinic. The Hyrax was activated twice a day. Patients were followed up weekly until clinical observation of molar transverse relation overcorrection. The clinical favorable accomplishment of RME was confirmed by the presence of the interincisal diastema (Fig 1).^3^ The device was maintained for retention, and patients were subsequently treated with fixed orthodontic appliances.

CBCT scans were obtained with an i-CAT tomography scanner (Imaging Sciences International, Hatfield, PA, USA) before the RME. On the images, the MS was evaluated with respect to the density ratio (MSD), maturation stage (MSM), and measurements of palatal length, thickness, and sagittal area. Data from the CBCT with extended field of view were exported in DICOM format to Invivo Dental 5.1 (Anatomage, San Jose, CA, USA) and Dolphin 3D imaging (Dolphin Imaging Systems, Chatsworth, CA, USA) softwares.

**Figure 2:**
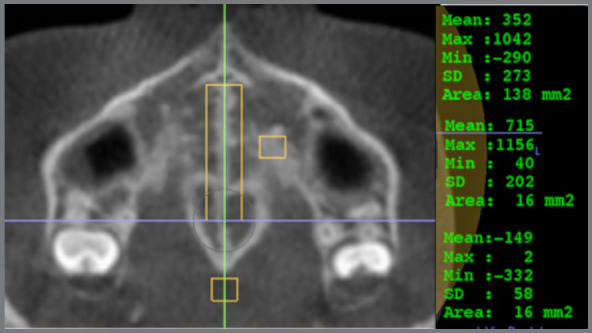
Regions used to determine MSD. The gray density of the palatal process and the soft palate was determined in a 4x4-mm area; the gray density of the suture was determined in a 6 mm-wide rectangle.

**Figure 3:**
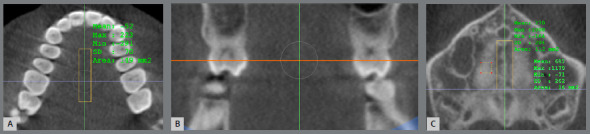
Determining the posterior limit of the rectangle of the suture. **A, B)** The largest diameter of the crown of tooth #16 in axial ( purple line ) and coronal ( orange line ) slices; **C**) The posterior border of the central rectangle in MS ( purple line ).

**Figure 4:**
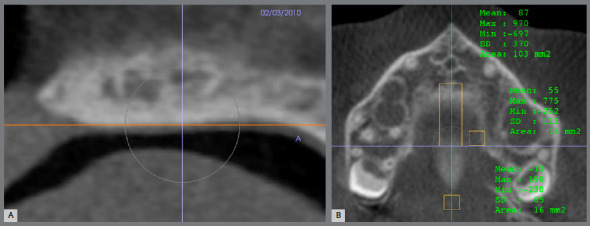
Density in the soft palate. **A)** Axial slice ( orange line ) positioned in the inferior limit of palate; **B)** 4 x 4-mm square in the center of soft palate.

The evaluation of MSD was performed in the InVivo software, based on the methodology described by Grunheid, Larson and Larson.^10^ The images were oriented; then, the density values ​​of the regions of interest were obtained (Fig 2). For the determination of the posterior border of the central rectangle in MS, the largest diameter of the crown of the first molars was used as reference and, in cases of asymmetry, tooth #16 was used as reference (Fig 3). The region of the suture and the palatine process were determined in the central axial slice of the hard palate. The inferior axial slice of the hard palate was used to set the soft palate rectangle by moving the axial line in the sagittal slice to the lower limit of the hard palate. Then the rectangle was positioned in the center of the soft palate in the axial slice (Fig 4). The mean values ​​of the density in each region were used to calculate MSD according to the equation below:^10^

$$MSD=\;\frac{Density{\;}_{suture\;}-\;Density{\;}_{soft\;palate\;}}{\;Density{\;}_{palatal\;process\;}-\;Density{\;}_{soft\;palate\;}}$$

The MSD ratio can range from 0 to 1, with lower values indicating less calcification, and higher values indicating greater calcification. 

After this assessment, patients were divided in groups of MSM stages (C, D and E) and in groups of lower density (MSD < 0.75) and higher density (MSD ≥ 0.75).

Evaluation of the MSM stage was performed in the axial slices of the pre-treatment scans using InVivo. Head positioning and slice acquisition were performed according to the steps described by Angelieri et al.^7^ These images were then organized in Microsoft Office - Power Point (2007; Microsoft, Redmond, Washington), with a black background, numbered, and with no identifying information such as name or age. Two observers, who were experienced in the evaluation of tomographic images and MSM analysis, classified each patient’s suture into stages A, B, C, D, or E.^7^

**Figure 5:**
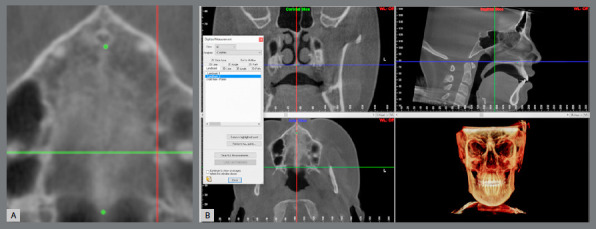
Points PNS and PF: **A)** selected in axial slice; **B)** confirmed in a sequence of slices.

**Figure 6:**
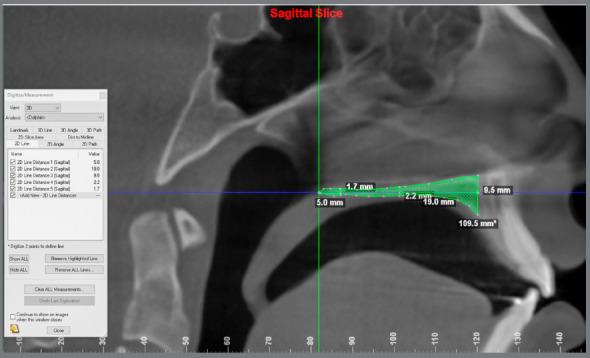
Palate measurements: length (19.0 mm), thickness (9.5 mm anterior; 2.2 mm intermediate; 1.7 mm posterior) and area (109.5 mm^2^).

The assessment of the length, thickness, and sagittal area of the palate was performed on the sagittal slice corresponding to the midsagittal plane of the scans, using Dolphin software. The images were oriented according to the palatal plane based on the orientation described in the methods above.^7^ ^,^ ^10^ Natural head position in all three planes of space (axial, sagittal and coronal) was obtained and, in the sagittal view, the patient’s head was positioned so that the anteroposterior long axis of the palate was parallel to the horizontal plane. The point posterior to the incisive foramen (PF) and the posterior nasal spine (PNS) were selected in the axial slice and checked in the other slices (Fig 5). The measurements were performed in the sagittal slice. Palatal length was determined by the horizontal distance between PF and PNS. The sagittal area was determined; and the thickness measurements were performed with the limits of the edges of the palate area (Fig 6). The anterior thickness intersected the PF point, the intermediate intercepted half the length of the palate, and the posterior one was located 5 mm anterior to the PNS. 

A single operator performed the linear and area measurements of the palate, and the density measurements. After repeating all measurements after a two-week interval, the calibration of the operator was tested with the intraclass correlation coefficient (ICC). Categorical variables were described quantitatively and by percentage of individuals in each category (stages of maturation and sex). Descriptive statistics of continuous variables (age and density) were provided. Normality of the data was tested with the Shapiro-Wilk test. The chi-square and Fischer’s exact test were used to compare differences in MSM and sex distribution; for comparison of age and MSD, the ANOVA with Tukey post-test was used between MSM groups, and the independent *t* test, between density groups.

## RESULTS

Twenty-six patients were included in the sample (6 males and 20 females). Patients’ age ranged from 14 to 28 years (mean of 16.42 years), and only the C, D, and E stages of MSM were represented. With respect to MSD, the patients were divided into two groups of 13 patients each, and characterized according to low or high density. The operator was calibrated (ICC ranged from 0.836 to 0.985). The linear and area measurements obtained from the sample were normally distributed.

When divided into two groups according to density, a significant difference was observed in relation to MSM stages (*p*= 0.003) and MSD (*p*< 0.001) (Table 1). 

**Table 1:** Sample characteristics, with patients divided into two groups, according to the midpalatal density ratio (MSM stages distribution; gender distribution; age, and density mean and standard deviation, SD) and *p*-value (significance) of chi-square test for differences in distribution, and independent *t* test for differences in mean.

MSM stage	Lower density (n=13)		Higher density (n=13)		Significance
	n	%	n	%	
C	4	30.8	1	7.7	0.003*
D	9	69.2	4	30.8	
E	0	0	8	61.5	
Sex					
Male	5	38.5	1	7.7	0.063
Female	8	61.5	12	92.3	
	Mean (SD)	Range	Mean (SD)	Range	
Age	17.38 (2.53)	14-23	15.46 (3.86)	14-28	0.147
Density ratio	0.60 (0.07)	0.47-0.72	0.82 (0.06)	0.75-1.0	<0.001**

* *p* < 0.01; **p < 0.001.

Regarding the different stages of MSM (Table 2), MSD and patients’ age were significantly different (*p*< 0.05). MSD increased progressively from stages C to E, with fusion of the suture. Age decreased with progressing stages of MSM.

**Table 2:** Sample characteristics, with patients divided in MSM stages (gender distribution; age and density mean and standard deviation, SD) and *p*-value (significance) of Fisher’s exact test for differences in distribution, and ANOVA for differences in mean.

	Stage C (n=5)		Stage D (n=13)		Stage E (n=8)		Significance
	n	%	n	%	n	%	
Male	2	40	4	30.8	0	0	0.162
Female	3	60	9	69.2	8	100	
	Mean (SD)	Range	Mean (SD)	Range	Mean (SD)	Range	
Age	19.4 (5.07)	16-28	16.38 (2.87)	14-23	14.62 (1.06)	14-17	0.036*
Density	0.62 (0.12)	0.50-0.84	0.67 (0.10)	0.47-0.83	0.83 (0.08)	0.75-1.0	0.002**

*p < 0.05; **p < 0.01.

The length of the palate was not significantly different between the MSM and MSD groups (*p*> 0.05) (Tables 3 and 4). With respect to thickness, there was a statistically significant difference (*p*< 0.05) in the intermediate and posterior regions of the palate at the different stages, with the more advanced stages (D and E) tending to be thinner, which may indicate that the disjunction occurs in stages D and E when the patient has a smaller palate thickness (Table 3). The area presented statistically significant difference for the different MSM (*p*= 0.01) and MSD groups (*p*< 0.05) (Tables 3 and 4). Since length was not significant and the areas observed in stages D and E were smaller, thickness of the palate may have an important influence (except in the anterior region).

**Table 3:** Mean and standard deviation (SD) of palate measurements for each midpalatal suture maturation group (according to the stages), and *p*-value (significance) of ANOVA and Tukey’s *post-hoc* test applied for intergroup differences.

	Stage C (n=5)		Stage D (n=13)		Stage E (n=8)		Significance
	Mean (SD)	Range	Mean (SD)	Range	Mean (SD)	Range	
Length (mm)	34.82 (1.53)	32.20-36.00	35.72 (2.14)	31.50-39.90	34.33 (2.21)	31.10-37.10	0.328
Thickness (mm)							
anterior	9.84 (2.01)	7.90-13.20	9.73 (2.21)	5.00-12.70	8.43 (2.54)	4.60-13.00	0.406
intermediate	6.02 (2.23)^A^	3.70-9.60	3.40 (1.07)^B^	1.60-5.30	3.32 (0.90)^B^	2.10-4.80	0.002**
posterior	4.60 (1.91)^A^	3.10-7.90	2.68 (0.67)^B^	1.70-4.50	3.18 (1.22)^AB^	1.90-5.60	0.016*
Area (mm^2^)	196.30 (54.66)^A^	147.10-284.10	140.23 (34.42)^B^	94.10-217.50	124.42 (33.11)^B^	82.70-187.70	0.01*

A,B different superscript letters means statistically significant difference in the same line; *p< 0.05; **p < 0.01.

**Table 4:** Mean and standard deviation (SD) of palate measurements for each midpalatal suture density group and p-value (significance) of independent *t* test applied for intergroup differences.

	Lower density (n=13)		Higher density (n=13)		Significance
	Mean (SD)	Range	Mean (SD)	Range	
Length (mm)	35.57 (2.18)	31.50-39.90	34.66 (1.97)	31.10-37.10	0.277
Thickness (mm)					
anterior	10.19 (1.84)	7.40-13.20	8.52 (2.43)	4.60-13.00	0.061
intermediate	4.49 (1.81)	2.50-9.60	3.26 (1.23)	1.60-6.20	0.056
posterior	3.41 (1.60)	1.70-7.90	3.00 (0.99)	1.90-5.60	0.435
Area (mm^2^)	166.72 (45.38)	118.50-284.10	125.57 (35.28)	82.70-201.30	0.016*

*p < 0.05.

## DISCUSSION

RME is the most frequently chosen treatment in cases of maxillary atresia, with well-established benefits in growing patients.^1^ Late skeletal growth of the patient is proportional with less strength to promote opening of the MS, compared to individuals with earlier growth.^3^ ^,^ ^14^ Some methods have been proposed to individually assess patients and predict response to RME, based on analysis of maturation stages^9^ and density ratio of MS.^10^ However, information from a recently published systematic review indicates that evidence is still weak.^15^

The developmental stages of the MS have been defined histologically and divided into infantile, juvenile, and adolescent periods; in the third stage, MS separation is not possible without fracture occurring in the areas of interdigitation.^17^ Angelieri et al.^7^ consider the sutures in stages D and E to have fused partially or completely, and surgically-assisted rapid palatal expansion (SARPE) could then be considered.^9^ ^,^ ^12^ Tomographic studies of MSD have provided information about resistance to RME,^18^ and changes in MS before and after RME.^19^ ^,^ ^20^ The results indicate that a lower suture density is directly related to a clinical favorable accomplishment of the expansion.^10^ ^,^ ^18^ ^,^ ^20^

In the present sample, it was verified that participants’ mean age was significantly different among MSM stages, similarly to a previous study.^21^Additionally, in a published study^22^ with 16 to 20-year-old participants, MSM stages C, D and E were the most often observed, similar to the present sample. Angelieri et al.^23^ reported that chronological age could also be considered a viable alternative to predict suture maturation. In the present study, the mean age decreased with progressing stages of MSM, indicating that RME may probably be better accomplished in older patients if they are still in earlier stages of fusion. With respect to the two MSD groups defined in this study (low and high density), no significant difference was found for age, which is similar to previous studies.^10^ ^,^ ^18^

The sample in this study was predominantly female, and no significant difference was found regarding patient sex in different MSM or MSD groups, although the composition of the high-density and the last maturation stage groups were more than 90% and 100% female, respectively. The same was observed in other studies, where 77.2% of the patients in the more advanced MSM stages were female,^12^ 100% of the patients in stage E were female,^7^ or the percentage of female, separated by age (16-20 years), in stage E was higher than for male.^22^

The MSD was significantly different between MSM stages, increasing from stage C to E. This indicates that the more advanced maturation stages present a higher-density suture. When the suture is not calcified, it is similar to the gray levels at the density of the soft palate. As progression of suture closure advances, some bony spicules begin to appear, and calcified and non-calcified areas are visible. As a consequence, the density increases, which means that the gray levels in the suture are approaching that of the palatine process (cortical bone), until there is fusion of the suture.^10^

A significant difference between average MSM was observed when the patients were divided in groups of high and low MSD. In the low-density group, all individuals were in stages C and D; while in the high-density group, more than 60% of the patients were in stage E. Patients older than 13 years in stages A, B, or C of MSM may have favorable prognosis for RME,^9^ ^,^ ^12^ despite that in stage C the skeletal response is lower than in the previous stages. Nevertheless, other authors^10^ reported the correlation between MSM and clinical skeletal measures after RME as negative and not significant, and they considered that density better predicted the response to RME.

The thickness of the palate might interfere in the response to RME, and thinner palates probably have less resistance to the forces of the treatment.^12^ In the present study, palate thickness was smaller in individuals who were in the final stages of MSM and in the high density group. These findings suggest that RME may present better prognosis in individuals in the final stages of MSM if the palate is thinner. The significant results found in the palate area using the sagittal slice for measurement were probably due to thickness differences, which corroborates the idea that thickness must be considered in the diagnosis, in addition to the maturation stages and/or density of MS. These variables (MSM, MSD and palatal measurements) can help the orthodontist decide about trying RME or indicating SARPE. Therefore, MSM stages A, B or C, and/or with low suture density values would indicate RME; and thin palates could indicate trying RME even in older patients in MSM stages D or E, and/or with high density values. SARPE would be recommended in patients in MSM stages D or E with high density values and thick palates. However, these findings should be confirmed in RCTs.

Patients’ division in groups of MSM stages was more informative than division in groups of density when the length, thickness and the area of the palate were evaluated, at least in the present sample, even though there were fewer patients in each stage. Differences in age were observed, which shows that the MSM stages allow better defined classification of the characteristics of palate and range of age, whereas differences in groups of MSD were not so marked. Nonetheless, density might be a predictor of RME outcomes; however, if groups are divided by density, characteristics of palate and the age of patients may not be so well defined, since there may be significant individual changes in the MSD. 

The present findings and other recently published articles^9^ ^,^ ^12^ about individual analysis of MSM showed that the CBCT can be used in cases of maxillary atresia with RME questionable prognosis. The evaluation of MSM stages, density, and thickness of palate provides valuable information in patients older than 14 years of age. The radiation dose must be as low as reasonably achievable (ALARA principle); therefore, CBCT with reduced field of view should be requested, which can reduce the radiation dose to the patient and present important information to treatment planning.^10^ ^,^ ^24^ ^,^ ^25^

It is important to mention that, in addition to midpalatal suture, other structures - such as internasal, maxillonasal, frontomaxillary, frontonasal, zygomaticomaxillary, zygomaticotemporal, and zygomaticofrontal sutures and spheno-occipital synchondrosis - can be affected by RME, and may also be used in pre-expansion analysis, in order to determine the best treatment for each patient.^26^ ^-^ ^28^

The clinical relevance of this study is that, although midpalatal suture opening during orthodontic treatment with rapid maxillary expansion is frequent and may reach 12 to 52 percent of the total screw expansion,^29^ failures may occur and, although SARPE has proven long-term stability,^30^ it is also a rather invasive procedure.

One of the limitations of the present study was its retrospective nature. Ideally, the study should be conducted as a randomized clinical trial with patients treated by the same orthodontist. The absence of an occlusal radiograph or CBCT after the RME is another limitation of present study, because these exams could have provided information about the proportion of skeletal and dental results. Further studies are necessary to corroborate the present findings. 

## CONCLUSIONS

In 14-year old or older patients submitted to rapid maxillary expansion with a clinical favorable accomplishment, smaller sagittal area was observed in patients with high midpalatal suture density or suture maturation in stages showing partial or complete fusion. A tomographic individual analysis of midpalatal suture characteristics is recommended in older adolescents and young adults, to consider the possibility of conservative and less invasive treatment.
